# Vancomycin Antibiotic Prophylaxis Compared to Cefazolin Increases Risk of Surgical Site Infection Following Spine Surgery

**DOI:** 10.1177/21925682251341833

**Published:** 2025-05-07

**Authors:** Brandon J. Herrington, Jennifer C. Urquhart, Parham Rasoulinejad, Fawaz Siddiqi, Kevin Gurr, Christopher S. Bailey

**Affiliations:** 1 Combined Orthopaedic and Neurosurgical Spine Program, 10033 London Health Sciences Centre, London, ON, Canada; 2 10033 London Health Sciences Centre Research Institute, London, ON, Canada; 3 Department of Surgery, Division of Orthopaedics, Schulich School of Medicine and Dentistry, 70384 University of Western Ontario, London, ON, Canada

**Keywords:** spine, lumbar, thoracic, surgery, fusion, infection, vancomycin, antibiotic, prophylaxis

## Abstract

**Study Design:**

Retrospective analysis of randomized controlled trial.

**Objectives:**

Surgical site infection (SSI) after spine surgery has severe negative health and financial consequences. Surgical antibiotic prophylaxis (SAP) is a routinely used method to prevent SSIs in the spine patient population. The most commonly used antibiotic is cefazolin, with vancomycin often being substituted in the case of penicillin or cephalosporin allergy. Vancomycin as SAP has been associated with increased SSI in the joint replacement literature, but this is not yet well defined in the spinal surgery population. The purpose of this study was to determine whether vancomycin SAP compared to cefazolin SAP is associated with increased risk of SSI.

**Methods:**

535 patients, aged 16 years or older, underwent elective multi-level open posterior spinal fusion surgery at the thoracic, thoracolumbar, or lumbar levels. Demographic and operative characteristics as well as post-operative outcomes were compared between the following groups: (1) noninfected-cefazolin, (2) noninfected-vancomycin, (3) infected-cefazolin, and (4) infected-vancomycin. Primary outcomes were superficial and complicated (deep and organ/space) infections.

**Results:**

The following risk factors for SSI were identified in a logistic regression analysis: vancomycin (OR 2.498, 95% CI, 1.085-5.73, *P* = 0.031), increasing operating time (OR 1.006, 95% CI, 1.001-1.010 *P* = 0.010), weight (OR 1.020, 95% CI 1.006-1.034, *P* = 0.005), revision procedure (OR 2.343, 95% CI 1.283-4.277, *P* = 0.006), and depression (OR 2.366, 95% CI 1.284-4.360, *P* = 0.006).

**Conclusions:**

In open posterior approach spinal fusion surgery, vancomycin SAP is associated with increased risk of infection compared to cefazolin SAP.

## Introduction

Surgical site infection (SSI) following spine surgery not only extends hospital length of stay and necessitates additional surgery and/or prolonged antibiotic treatment, but also increases morbidity, mortality, and negatively impacts quality-of-life outcomes.^1,2^

One commonly used method to prevent SSIs is with surgical antibiotic prophylaxis (SAP).^3^ In general, an appropriate SAP agent should be active against those organisms most likely to cause an infection at the surgical site, and they should be given at an appropriate time and dose to ensure adequate concentration of antibiotic at the surgical site throughout the operation.^4^

In the setting of spine surgery, cefazolin is the most commonly used drug for SAP.^5,6^ It’s typically dosed at 2g within 60 minutes of surgical incision for patients <120 kg, and at 3g for patients >120 kg^4^. Cefazolin is effective as an agent for SAP for spine surgery,^7^ but sometimes alternative antibiotics are indicated as a replacement.

One of the most often administered alternative SAP agents is vancomycin.^8^ Typically, vancomycin is used when a patient has a severe allergy to beta-lactams,^9^ or when MRSA colonization is present.^10^

Recently, we published a randomized controlled trial (RCT) comparing 24 hours of post-operative SAP to 72 hours.^1^ This RCT found no difference in infection rates between the 2 different treatment methods. However, the infection rate in this study was relatively high. Given previous literature demonstrating increased risk of SSI with vancomycin SAP in patients undergoing total joint arthroplasty,^11,12^ we sought to determine whether vancomycin increases the risk of SSI when compared to cefazolin in this population of patients undergoing multilevel spinal fusion. Our hypothesis is that when vancomycin SAP is used instead of cefazolin SAP, risk of SSI increases.

## Methods

### Study Setting and Design

This is a retrospective analysis of prospectively collected data from a consecutive series of patients enrolled in a previously completed RCT that compared 2 postoperative antibiotic treatment strategies: standard preoperative SAP with cefazolin or vancomycin and (1) 24 hours of postoperative antibiotics, or (2) 72 hours of postoperative antibiotics (to provide an additional 24 hours antibiotic coverage following postoperative drain removal on postoperative day 2).^1^

### Patient Population

Patients were enrolled between October 2011 and April 2016 at a single centre. The details of the primary trial have been published previously.^1,13^ Briefly, patients were randomly assigned by 1:1 allocation to duration of antibiotics for 24 hours after the operation or for 24 hours after drain removal (72 hours after the operation). The initial trial randomization was stratified by surgeon and presence or absence of diabetes. Drains were discontinued on the second postoperative day. The duration of antibiotic administration was not blinded. The operative technique used was chosen according to the surgeon’s discretion on a patient-by-patient basis. All patients received pre-operative antibiotic prophylaxis. This included either 2g of cefazolin intravenously 1 hour to no later than 15 minutes prior to incision and a second dose of cefazolin intraoperatively at the 4-hour mark (if necessary), or 1g of vancomycin intravenously within 1 hour of incision if the patient was allergic to penicillin or cephalosporin.^14^ All patients had a closed-suction drain used (10-French Jackson Pratt Wound drain, Cardinal Health). Postoperatively, patients received 1g of cefazolin every 8 hours or 1g of vancomycin every 12 hours, according to their randomization. Drain removal was standardized to approximately 48 hours postoperatively.

### Inclusion and Exclusion Criteria

For the present study, patients were included if they were 16 years of age and older and underwent elective, multi-level open posterior spinal fusion surgery at the thoracic, thoracolumbar, or lumbar levels with placement of a closed-suction drain. Exclusion criteria were known hypersensitivity to both cefazolin and vancomycin; renal function impairment (creatinine level of >100 μmol/L); antibiotic therapy for concomitant infection; surgery for spinal tumor or spinal trauma; surgery for spinal infection; pregnancy, concomitant corticosteroid therapy, and permanent residence >5 hours driving distance from surgical geographic location. In the original trial, 552 patients were enrolled. However, in the present study, patients that had deformity (n = 12) or multilevel decompression with no fusion (n = 5) were excluded.^1,13^ Therefore, 535 patients were included. Approval was obtained from the institutional ethics board (#100561). Written informed consent was obtained from all participants.

### Outcome Measures

Primary outcome was a complicated infection (deep or organ/space) or a superficial infection within 1 year of the surgical procedure. Superficial, deep, and organ/space surgical site infections were defined according to the U.S. Centres for Disease Control and Prevention criteria.^15^ Demographic variables evaluated as risk factors for surgical site infection were age, gender, body mass index, diabetes, anemia or other blood disease, hypertension, cerebrovascular disease, osteoarthritis and inflammatory arthritis, heart disease, lung disease, gastrointestinal disease, liver disease, history of cancer, smoking history, depression, pre-operative Medical Outcomes Study-12 item Short-Form General Health Survey (SF12) mental component summary score (MCS) and physical component summary score (PCS) and Oswestry Disability Index (ODI), and diagnosis.^16^ BMI was dichotomized to ≥30 kg/m^2^ as this is a cut-point for obesity. Operative variables evaluated as risk factors included duration of postoperative antibiotics (randomized to 24 hours or 72 hours of postoperative antibiotics in the original RCT), type of procedure (interbody fusion or instrumented fusion), ASA classification, antibiotic type (vancomycin vs cefazolin), revision procedure, number of operated levels, operating time, and estimated blood loss. Other factors associated with infection that were identified for collection a priori included the season that the procedure was performed, number of surgical assistants, number of scrub events, and postoperative day 1 hemoglobin level.

### Statistical Analysis

Data analysis was performed using SPSS Statistics version 27 (SPSS Inc., Chicago, IL, USA). The demographic and operative characteristics as well as post-operative outcomes were compared between the following groups: (1) noninfected-cefazolin, (2) noninfected-vancomycin, (3) infected-cefazolin, and (4) infected-vancomycin. Between group comparisons were made using a one-way ANOVA for continuous parametric variables or Kruskal-Wallis test for continuous nonparametric variables. Comparisons for categorical variables were made using the Chi-square test or the Fisher’s exact test. Multiple comparisons were conducted using a Bonferroni post hoc test or an independent samples test for proportions.

To identify the risk factors associated with surgical site infection, type of antibiotic as well as variables that yielded a *P* value of ≤0.05 in univariate analysis were included in the multivariate analysis. Variables were also included if they were clinically important by the authors’ consensus. Collinearity was evaluated by calculation of tolerance. Multivariate backward stepwise, conditional, logistic regression was used to identify the best combination of variables that predict having a surgical site infection vs not having an infection. Individual predictor variables were eliminated in a backward fashion if the corresponding *P* value was ≥0.10. Cases with missing data were excluded from the model. The discriminative ability was assessed using ROC curves and by comparing the predicted vs the observed surgical site infections by area under the curve.

## Results

### Patient Demographics

Baseline patient characteristics are detailed in Table 1. 535 total patients were included in the analysis with 429 in the noninfected-cefazolin group, 27 in the noninfected-vancomycin group, 69 in the infected-cefazolin group, and 10 in the infected-vancomycin group. No between group differences were noted for age, sex, height, primary diagnosis, smoking status, baseline disability (ODI and SF12 scores), or medical comorbidities except for depression (*P* = 0.009). Between group differences were noted for weight (*P* = 0.004), BMI ≥30 kg/m^2^ (*P* = 0.002), and revision procedure (*P* = 0.001). Patients in the infected-cefazolin group weighed more than patients in the noninfected-cefazolin group (91 ± 19 kg vs 83 ± 18 kg, *P* = 0.002). Weight was not significantly different between the infected-vancomycin and noninfected-vancomycin groups (85 ± 16 kg vs 81 ± 20 kg, *P* = 1.000). However, more patients in the infected-vancomycin group had a BMI ≥30 kg/m^2^ compared to those treated with vancomycin who did not develop an infection (80% vs 37%, *P* = 0.020). More patients in the infected-cefazolin group had a BMI ≥30 kg/m^2^ compared to patients in the noninfected-cefazolin group (62% vs 43%, *P* = 0.003). More patients in the infected-cefazolin group were undergoing a revision procedure compared to those in the noninfected-cefazolin group (29% vs 15%, *P* < 0.001) whereas a similar proportion of patients in the infected-vancomycin and noninfected-vancomycin groups were undergoing a revision procedure (40% vs 33%, *P* = 0.706). More patients in the infected groups had preoperative depression (infected-cefazolin 29%, vs noninfected-cefazolin 16%, *P* = 0.009, and infected-vancomycin 30% vs noninfected-vancomycin 4%, *P* = 0.022).

**Table 1.** Patient-Specific Risk Factors Stratified by Antibiotic Type and the Presence of Infection.

Variable	Noninfected		Infected		*P*-Value
	Cefazolin n = 429	Vancomycin n = 27	Cefazolin n = 69	Vancomycin n = 10	
	Value	Value	Value	Value	
Age, years, mean ± SD	59.3 ±14.3	61.3 ± 14.7	57.7 ± 13.2	67.6 ± 12.5	0.183
Sex, male, n (%)	207 (48.3)	10 (37.0)	31 (44.9)	2 (20.0)	0.223
BMI ≥30 kg/m^2^, n (%)	184 (42.9)	10 (37.0)	43 (62.3)	8 (80.0)	**0.002**
Weight, kg					
Mean ± SD	82.5 ± 18.0	80.9 ± 19.6	90.9 ± 18.8*	84.6 ± 16.3	**0.004**
Median (min-max)	82 (45-152)	79 (43-141)	93 (51-129)	85 (59-104)	
Weight ≥66 kg, n (%)	340 (79.3)	23 (85.2)	61 (88.4)	8 (80.0)	0.313
Height, m					
Mean ± SD	1.7 ± 0.1	1.7 ± 0.1	1.7 ± 0.1	1.6 ± 0.1	0.187
Median (min-max)	1.7 (1.2-2.1)	1.7 (1.5-1.9)	1.7 (1.3-1.9)	1.6 (1.5-1.8)	
Diabetes, n (%)	65 (15.2)	7 (25.9)	15 (21.7)	3 (30.0)	0.180
Smoker, n/total no^b^ (%)	103/402 (25.6)	5/25 (20.0)	16/61 (26.2)	2 /10(20.0)	0.903
Primary diagnoses, n (%)					0.599
Disc herniation^a^	88 (20.5)	3 (11.1)	15 (21.7)	1 (10.0)	
Stenosis	64 (14.9)	4 (14.8)	9 (13.0)	2 (20.0)	
Stenosis + degen. Scoliosis	15 (3.5)	0 (0.0)	3 (4.3)	0 (0.0)	
Degen. Spondylolisthesis	180 (42.0)	11 (40.7)	30 (43.5)	7 (70.0)	
Isthmic spondylolisthesis	82 (19.1)	9 (33.3)	12 (17.4)	0 (0.0)	
Revision procedure, n (%)	63 (14.7)	9 (33.3)	20 (29.0)	4 (40.0)	**0.001**
Comorbidity^c^, n (%)					
None	87 (20.3)	2 (7.4)	9 (13.0)	2 (20.0)	0.218
Anemia or other blood disease	29 (6.8)	0 (0.0)	4 (5.8)	1 (10.0)	0.530
Hypertension	61 (14.2)	3 (11.1)	11 (15.9)	2 (20.0)	0.888
Cerebrovascular disease	12 (2.8)	0 (0.0)	3 (4.3)	0 (0.0)	0.643
Joint disease (osteoarthritis)	85 (19.8)	6 (22.2)	14 (20.3)	2 (20.0)	0.992
Heart disease	17 (4.0)	0 (0.0)	1 (1.4)	0 (0.0)	0.468
Lung disease	35 (8.2)	4 (14.8)	12 (17.4)	0 (0.0)	0.809
Gastrointestinal disease	10 (2.3)	2 (7.4)	2 (2.9)	0 (0.0)	0.414
Liver disease (mild/moderate)	7 (1.6)	0 (0.0)	0 (0.0)	0 (0.0)	0.625
History of cancer	20 (4.7)	1 (3.7)	5 (7.2)	1 (10.0)	0.696
Other	8 (1.9)	0 (0.0)	2 (2.9)	0 (0.0)	0.776
Depression	69 (16.1)	1 (3.7)	20 (29.0)	3 (30.0)	**0.009**
Inflammatory arthritis	2 (0.5)	0 (0.0)	1 (1.4)	0 (0.0)	0.740
Unknown	64 (14.9)	4 (14.8)	12 (17.4)	0 (0.0)	0.556
ODI, n, mean ± SD	358, 47.9±16.3	21, 47.2 10.9	59, 51.2 ± 16.6	10, 49.8 ± 13.0	0.495
SF12 PCS, n, mean ± SD	351, 28.5 ± 6.7	21, 28.3 ± 7.4	52, 26.7 ± 6.2	9, 27.5 ± 9.9	0.161
SF12 MCS, n, mean ± SD	351, 44.1 ± 11.0	21, 44.8 ± 8.4	52, 40.7 ± 12.6	9, 40.5 ± 13.0	0.323

Abbreviations: BMI = body mass index, ODI = Oswestry Disability Index; SF12 = Medical Outcomes Study-12 item Short-Form General Health Survey, PCS = physical component summary score; MCS = mental component summary score. *P < 0.05 cefazolin non-infected vs cefazolin infected.

^a^Includes recurrent, intra-foraminal, and multilevel disc herniation as well as and central protrusions producing spinal stenosis.

^b^Different denominators indicate available data.

^c^Patients could have more than 1 comorbidiy.

### Intra- and Peri-Operative Factors

Statistically significant between group differences were noted in number of levels operated (*P* = 0.010) and operating time (*P* = 0.043; Table 2). Post hoc analysis showed that, on average, patients in the infected-cefazolin group had a longer operating time than patients in the noninfected-cefazolin group by 26 minutes (*P* = 0.011). The infected-cefazolin group also had more levels operated on compared to those in the noninfected-cefazolin group (1.8 ± 1.1 levels vs 1.4 ± 0.9 levels, *P* = 0.002). The difference in operating time and operative levels were not different between infected-vancomycin and noninfected-vancomycin groups.

**Table 2.** Comparison of Intra-Operative and Peri-Operative Risk Factors Stratified by Antibiotic Type and the Presence of Infection.

Variable	Noninfected		Infected		*P*-Value
	Cefazolin n = 429	Vancomycin n = 27	Cefazolin n = 69	Vancomycin n = 10	
Antibiotic duration, n (%)					0.795
24 hours	216 (50.3)	12 (44.4)	37 (53.6)	6 (60.0)	
72 hours	213 (49.7)	15 (55.6)	32 (46.4)	4 (40.0)	
Surgical procedure, n (%)					0.418
Interbody fusion	359 (83.7)	23 (85.2)	63 (91.3)	8 (80.0)	
Instrumented fusion	70 (16.3)	4 (14.8)	6 (8.7)	2 (20.0)	
Segmental levels operated, n (%)					**0.010**
Mean ± SD	1.4 ± 0.9	1.7 ± 1.0	1.8 ± 1.1*	1.6 ± 0.8	
Median (min-max)	1 (1-13)	1 (1-5)	1 (1-8)	1 (1-3)	
ASA ≥3, n (%)	262 (61.1)	18 (66.7)	50 (72.5)	9 (90.0)	0.085
Operating time, (min)					**0.043**
Mean ± SD	205 ± 55	220 ± 54	231 ± 78*	196 ± 38	
Median (min-max)	204 (58-391)	217 (120-386)	211 (71-630)	187 (150-260)	
Blood transfusion, n (%)	31 (7.2)	3 (11.1)	6 (8.7)	2 (20.0)	0.434
Estimated blood loss, (mL)	406	25	62	10	0.083
Mean ± SD	659 ± 385	766 ± 456	819 ± 588	660 ± 292	
Median (range)	600 (100-3000)	690 (200-2000)	700 (50-3500)	500 (350-1200)	
Spring/summer season, n (%)	204 (47.6)	12 (44.4)	33 (47.8)	6 (60.0)	0.867
No. of surgical assistants, n (%)					0.790
Median (min-max)	2 (1-5)	2 (1-3)	2 (1-5)	2 (1-3)	
No. of scrub events					0.490
Median (min-max)	4 (1-6)	4 (2-6)	4 (1-6)	5 (3-6)	
Hemoglobin postop day 1, g/L					0.413
n	422	27	69	10	
Mean ± SD	106 ± 14	103 ± 15	108 ± 15	102 ± 14	
Median (min-max)	105 (73-163)	101 (73-141)	106 (74-148)	104 (84-127)	

*P < 0.05 cefazolin non-infected vs cefazolin infected.

### Infection Details

Superficial SSIs made up a majority of total SSIs in the infected-cefazolin group and a minority in the infected-vancomycin group (64% vs 40%, *P* = 0.001), while the majority of SSIs were complicated (deep or organ/space) in the infected-vancomycin group compared to the infected-cefazolin group (60% vs 36%, *P* = 0.001). The time to infection was similar between cefazolin and vancomycin groups (median 15 days vs 16.5 days; *P* = 0.275). Further infection details are found in Table 3.

**Table 3.** Infection Details.

Variable	Infected		*P* Value
	Cefazolin n = 69	Vancomycin n = 10	
Superficial SSI	44/69 (63.8)	4/10 (40.0)	**0.001**
Complicated SSI (deep + organ space)	25/69 (36.2)	6/10 (60.0)	**0.001**
Type of organism in complicated SSI, n (%)			
Negative	4/25	2/6	
Staphylococcus Aureus	13/25	2/6	
Coagulase negative staphylococcus	1/25	0/6	
*Enterococcus faecalis*	1/25	0/6	
Streptococcus species	2/25	1/6	
*Enterobacter cloacae*	1/25	1/6	
*Escherichia coli*	2/25	0/6	
Coagulase negative staphylococcus + *P. acnes*	1/25	0/6	
Time to SSI, days			0.275
Mean ± SD	19.3 ± 22.6	46.4 ± 90.4	
Median (min-max)	15.0 (3-178)	16.5 (11-303)*	

SSI, superficial, deep and organ/space SSI defined by the Centre for Disease Control and Prevention occurring within 1 year of surgery. *includes 1 organ space infection – adjacent level discitis treated at 303 days after surgery. Time to SSI *P*-value was derived using Mann-Whitney U Test.

### Post-Operative Outcomes

Adjacent level condition, pseudoarthrosis, instrument-related adverse events, neurologic deterioration, and new onset pain were not different between groups (*P* < 0.05, Table 4). Patients in the infected-cefazolin group were more likely to have a cerebrospinal fluid leak or hematoma than any other group (*P* < 0.05, respectively). Patients in the infected groups had more wound dehiscence (infected-cefazolin 18.8% vs noninfected-cefazolin 0.5%, and infected-vancomycin 20% vs noninfected-vancomycin 4%, *P* = 0.001), more post-discharge emergency room visits (infected-cefazolin 12% vs noninfected-cefazolin 3%, and infected-vancomycin 10% vs noninfected-vancomycin 0%, *P* = 0.003), and more additional surgeries (infected-cefazolin 36% vs noninfected-cefazolin 3%, infected-vancomycin 50% vs noninfected-vancomycin 4%, *P* = 0.001) as a result of the infection compared to patients in the noninfected groups. The rate of emergency room visits and additional surgeries did not differ between infected-cefazolin and infected-vancomycin groups (*P* > 0.05 for both comparisons). The mortality rate was 10% in the infected-vancomycin group (n = 1) and less than 4% in the other groups (*P* = 0.007). All deaths were unrelated to infection.

**Table 4.** Comparison of Post-operative Outcomes Stratified by Antibiotic Type and the Presence of Infection.

Variable	Noninfected		Infected		*P*-Value
	Cefazolin n = 429	Vancomycin n = 27	Cefazolin n = 69	Vancomycin n = 10	
Adverse events, n (%)^a^					
Adjacent level condition	4 (0.9)	0 (0.0)	3 (4.3)	0 (0.0)	0.117
Pseudoarthrosis	2 (0.5)	1 (3.7)	0 (0.0)	0 (0.0)	0.151
Instrument/Fixation/Malposition	4 (0.9)	0 (0.0)	1 (1.4)	0 (0.0)	0.909
Myocardial infarction	1 (0.2)	0 (0.0)	1 (1.4)	0 (0.0)	0.474
Neurologic deterioration	4 (0.9)	1 (3.7)	0 (0.0)	0 (0.0)	0.394
New onset pain	18 (4.2)	2 (7.4)	5 (7.2)	1 (10.0)	0.530
Cerebrospinal fluid leak	2 (0.5)	0 (0.0)	5 (7.2)	0 (0.0)	**0.001**
Hematoma	6 (1.4)	0 (0.0)	5 (7.2)	0 (0.0)	**0.012**
Dehiscence	2 (0.5)	1 (3.7)	13 (18.8)	2 (20.0)	**0.001**
Other	5 (1.2)	0 (0.0)	1 (1.4)	0 (0.0)	0.920
Non-spinal infection, n (%)^b^	16 (3.8)	0 (0.0)	6 (8.9)	0 (0.0)	0.155
Intensive care unit admission, n (%)	2 (0.5)	0 (0.0)	2 (2.9)	0 (0.0)	0.169
Length of stay, days					0.115
Mean ± SD	4.0 ± 1.8	4.3 ±1.8	5.0 ± 5.5	8.7 ± 9.3	
Median (min-max)	4 (1-14)	4 (2-9)	4 (1-43)	5 (1-27)	
Post-discharge emergency room visit, n (%)	12 (2.8)	0 (0.0)	8 (11.6)	1 (10.0)	**0.003**
Additional surgery, n (%)^c^	14 (3.3)	1 (3.7)	25 (36.2)	5 (50.0)	**0.001**
Mortality^d^, n (%)	3 (0.7)	1 (3.7)	0 (0.0)	1 (10.0)	**0.007**

^a^Some patients had more than 1 adverse event.

^b^Requiring antibiotics, ie, urinary tract infection, sepsis, pneumonia.

^c^At least 1 additional surgery within 2 years of index surgery including irrigation & debridement.

^d^Deaths were not related to infection.

### Multivariable Results

The following risk factors for SSI were identified in a logistic regression analysis: vancomycin (OR 2.498, 95% CI 1.085-5.753, *P* = 0.031), increasing operating time in minutes (OR 1.006, 95% CI 1.001-1.010, *P* = 0.010), weight in kg (OR 1020, 95% CI 1.006-1.034, *P* = 0.005), revision procedure (OR 2.343, 95% CI 1.283-4.277, *P* = 0.006), and depression (OR 2.366, 95% CI 1.284-4.360, *P* = 0.006). These results are displayed in Table 5. Patients who received vancomycin prophylaxis had more than twice the odds of developing a SSI than those who received cefazolin. Similarly, those undergoing a revision procedure also faced double the odds of having an SSI. For every 5-kg increase in body weight, the odds of infection rose by 10%, while a 30-minute increase in operating time led to an 18% increase in the odds of infection. Due to 32 cases with missing data, the multivariate analysis included 503 cases. The Hosmer and Lemeshow test indicated a good fit to the data (Chi-square 9.721, *P* = 0.285). The area under the receiver operator characteristic curve was 0.710 ± 0.031, suggesting the model had moderate discriminative ability.

**Table 5.** Multivariate Logistic Regression Analysis: Significant Risk-Factors for Infection.

Risk-Factors	OR	95% CI	*P*-Value
Vancomycin	2.498	1.085 - 5.753	0.031
Operating time (min)	1.006	1.001 - 1.010	0.010
Weight (kg)	1.020	1.006 - 1.034	0.005
Revision procedure	2.343	1.283 - 4.277	0.006
Depression	2.366	1.284 - 4.360	0.006

Variables entered into the model: age, vancomycin, blood loss, operating time, weight (kg), revision procedure, ASA ≥3, diabetes, segmental levels operated, depression.

## Discussion

In the present retrospective analysis of an RCT of patients that underwent multilevel thoracolumbar surgery, vancomycin SAP was associated with almost a 2.5-fold higher risk of SSI than cefazolin SAP. These results echo previous findings within the orthopaedic literature but also have important distinctions. For instance, in a retrospective review of SAP agents in 622 patients who underwent elbow arthroplasty, vancomycin was associated with a 3.3 times greater risk of SSI when compared to cefazolin.^17^ Similarly, in a retrospective review of 7713 shoulder arthroplasties, when compared to cefazolin, a 2.3-fold greater risk of SSI was identified in patients who received vancomycin SAP.^18^ Large dataset reviews have found similar increased risk of SSI with vancomycin.^19,20^ However, other studies have shown similar rates of SSI between those receiving vancomycin or cefazolin SAP.^21^ Some have even demonstrated vancomycin to be advantageous in reducing SSI with drug resistant organisms.^22^ These conflicting results highlight 2 significant issues with vancomycin administration—dosing and timing.

In regards to timing of administration, Marigi et al re-examined a cohort of shoulder arthroplasty patients who had previously demonstrated higher rates of SSI with vancomycin SAP than with cefazolin.^18^ They showed that when patients received a complete dose of vancomycin—defined as at least 30 minutes of vancomycin infusion prior to incision—no difference was observed in rates of SSI compared to cefazolin.^23^ Hawn et al looked at a large dataset of patients who received various forms of antibiotic prophylaxis. In general, timing of antibiotic delivery was not a significant risk factor for SSI, but vancomycin compared to cefazolin was associated with higher risk of SSI, and it was administered 12 minutes earlier than cefazolin on average (40 vs 28 minutes).^20^ Since vancomycin is usually administered at a rate of 1 g/hour to avoid vancomycin flushing syndrome,^24^ this can potentially cause issues where the medication is not fully delivered prior to skin incision, and SAP delivery after skin incision has been linked to higher rates of SSI.^25^ In our analysis, a well-established protocol during the initial RCT ensured patients had completed their administration of vancomycin within 1 hour of skin incision. As such, in the present analysis, timing of vancomycin administration was not the cause of the increased risk of SSI with vancomycin SAP.

In this cohort of patients who underwent multilevel thoracolumbar fusion surgery, prophylactic vancomycin dosing was standardized to 1g for all patients.^10,26^ This “standardized” dose has been called into question, with more recent guidelines recommending weight-based dosing of 15 mg/kg^4^. Using these recommendations, Catanzano et al found that 69% of patients who received 1g vancomycin SAP prior to elective joint or spine surgery were underdosed.^27^ Similarly, when Kheir et al examined arthroplasty patients receiving vancomycin SAP, they showed that 94% of those who received 1g were underdosed, and that no patients who received the recommended 15 mg/kg dose developed a SSI.^28^ In the present cohort, the proportion of patients with BMI ≥30 kg/m^2^ was significantly different in the infected-vancomycin group compared to the noninfected-vancomycin group (80% vs 37%), which points to obesity as a significant risk factor for infection and potentially dosing issues as well. However, after examining patients with body weight ≥66 kg (the weight-based cutoff point for adequate therapeutic dosing using 1g vancomycin), the proportion of patients with weight ≥66 kg in the noninfected-vancomycin was actually higher than in the infected-vancomycin group (85% vs 80%), but this was not statistically significant. Although most patients in each group were underdosed according to newer weight-based dosing guidelines, weight ≥66 kg and weight on a continuous scale were not independent predictors of increased SSI when comparing the noninfected-vancomycin and infected-vancomycin groups.

With increased concern around infection with methicillin resistant organisms,^29^ some authors have demonstrated lower SSI rates by using both vancomycin and cefazolin together for SAP.^30^ This question was addressed by a recent large multicenter randomized controlled trial in a group of patients undergoing joint replacement surgery. The authors unfortunately did not find any reduction in SSI rates when patients received vancomycin and cefazolin SAP when compared to SAP with cefazolin and placebo.^31^ In addition, the combination of the 2 antibiotics has also been associated with higher incidence of acute kidney injury than either 1 of them alone.^32^

Although dosing and timing are the most cited reasons for differences in SSI between vancomycin and cefazolin SAP, 2 other less obvious characteristics may play a significant role in this discussion—molecular weight and charge.^33-35^ In a cohort of 54 patients who received prophylactic administration of either cefazolin, clindamycin, or vancomycin, and subsequently had their disc material analyzed following microdiscectomy, concentration of vancomycin in the disc material was significantly lower than that of cefazolin.^36^ The molecular weight of vancomycin is much greater than cefazolin (1449 g/mol vs 455 g/mol), and the authors postulated that this may have impacted the ability of vancomycin to penetrate the intervertebral disc. This is supported by other in vitro work demonstrating slower diffusion within an intervertebral disc with vancomycin compared to oxacillin (molecular weight = 401 g/mol)—despite the positive charge of vancomycin theoretically giving it an advantage over the negatively charged oxacillin (as well as cefazolin) to penetrate the negatively charged extracellular matrix of the disc.^37^

Recently, using gene expression analysis in a 2024 study, Hayles et al identified a cellular mechanism of *s. aureus* that allows it to modify its cell surface charge following attachment to medical grade titanium.^38^ In this novel study, prior to exposing *s. aureus* to titanium, both antibiotics had equal effectiveness. However, after a titanium surface was immersed in a *s. aureus* solution for 3 hours, only 20% of *s. aureus* survived upon treatment with cefazolin whereas 60% survived after treatment with vancomycin. When they extended this immersion to 24 hours, 50% of organisms survived with cefazolin while 90% survived after treatment of vancomycin. This study elucidates that not only are organisms more resistant to antibiotics quickly after exposure to titanium, but also that cefazolin is significantly more effective against titanium surface-associated *s. aureus* than vancomycin. This is particularly relevant to the fields of orthopaedic and spine surgery, as many of the instrumented materials are titanium-based.^39^ Of note, similar patterns of resistance have been shown with the positively charged antibiotic daptomycin.^40-42^

In the present analysis, patients only received vancomycin SAP when a documented beta-lactam allergy was identified. In a 2021 systematic review and meta-analysis of penicillin and cefazolin allergies, a true cefazolin allergy was identified in only 0.6% of patients with an unconfirmed penicillin allergy, and in only 3% of patients with a confirmed penicillin allergy. Furthermore, only 4.7% of patients allergic to cefazolin were also allergic to penicillin, suggesting low cross-reactivity.^43^ Given the increased risk of SSI associated with vancomycin, clinicians should ensure patients with suspected beta-lactam allergy are further investigated by an appropriate professional, as many of these individuals could still safely receive cefazolin. Indeed, in 1 2019 arthroplasty study, 97% of 2576 patients referred to an allergist for penicillin allergy prior to surgery were ultimately cleared for the use of cefazolin.^44^

Aside from vancomycin use, obesity, revision surgery, and depression were also found to be risk factors for SSI in our cohort. Obesity and revision surgery are well documented elsewhere as risk factors for SSI,^45,46^ and more evidence continues to emerge regarding risk of SSI with anxiety and depression.^47-49^ It’s thought that with depression, the increased risk of infection may be secondary to elevated cortisol levels suppressing the immune system and subsequently affecting wound healing rates, higher rates of smoking, or higher rates of nonadherence to medical recommendations in this population.^50,51^ In the present study, however, smoking was not an independent predictor of SSI.

### Limitations

A limitation of this study is selection bias. Due to patients receiving vancomycin only if they had an allergy to beta lactams, and more patients undergoing reoperation (which also carries a higher risk of infection) in the vancomycin group, the sample may not be fully representative of the broader population. Although a number of patient and operative risk factors including reoperation were controlled for in our multivariate analysis, we cannot control for any unknown factors that may have contributed to the increased risk of infection in patients receiving vancomycin. This is a limitation of the retrospective nature of the study. However, this data was gathered from a previous RCT where patients were recruited consecutively, which does strengthen the findings of the study and ultimately decrease the risk of that bias.

Our study population was limited to those who underwent instrumented fusion surgery only. This impacts our ability to generalize the results of the study to the population of spine patients undergoing surgery without instrumentation. Additionally, our study cohort consisted of patients who underwent open posterior approach surgery only. It’s possible that infection rates between those who received vancomycin and cefazolin SAP may be different in those who had more minimally invasive or non-posterior approaches. However, the majority of elective surgeries continue to be done via an open posterior approach, and continue to include instrumentation, which lends to the overall generalizability of this analysis.^52^

## Conclusions

In our cohort of patients who underwent posterior instrumented spine surgery, we identified a 2.5-fold increased risk of SSI in those who received vancomycin SAP compared to those who received cefazolin SAP, independent of dose and timing of administration. Clinicians should ensure patients with a suspected beta-lactam allergy receive appropriate pre-operative investigation into the nature of this allergy to prevent unnecessary administration of vancomycin in those who can ultimately tolerate cefazolin antibiotic prophylaxis.
